# Supplementary material for: Optimization of High-Density Fermentation Conditions for Saccharomycopsis fibuligera Y1402 through Response Surface Analysis
Source: Foods. 2024 May 16;13(10):1546. doi: 10.3390/foods13101546 (PMC11121647; doi:10.3390/foods13101546)
Supplement: Supplementary file 1 [file foods-13-01546-s001.zip › foods-2977575-supplementary.pdf]

Supplementary Tables

Supplementary Table S1. Levels of the variables and statistical analysis in PBD in OD_{560nm} for medium ingredient

Code	Variable	Level		Regression coefficient	*F* values	*P* values
		Low (-1)	High (1)			
X_1	Glucose concentration (g/L)	200	400	0.0733	38.43	0.004
X_2	Peptone concentration (g/L)	20	80	0.0150	0.83	0.418
X_3	Yeast extract concentration (g/L)	10	30	-0.0483	16.59	0.016
X_4	KH_2PO_4 concentration (g/L)	2	6	0.0550	13.29	0.024
X_5	$MgSO_4$ concentration (g/L)	0	0.8	0.0350	3.73	0.085
X_6	$CuSO_4$ concentration (g/L)	0	0.02	0.0500	4.87	0.068

Supplementary Table S2. Levels of the variables and statistical analysis in PBD in OD_{560nm} for fermentation condition

Code	Variable	Level		Regression coefficient	*F* values	*P* values
		Low (-1)	High (1)			
X_1	Initial pH	5	7	4.01	3.99	0.076
X_2	Inoculum size (v/v)	0.5	1.5	17.55	39.85	0.004
X_3	Rotation speed (r/min)	90	150	0.1325	3.82	0.078
X_4	Fermentation temperature (°C)	15	25	2.255	112.03	0.000
X_5	Fermentation time (d)	1	3	8.76	76.13	0.001

Supplementary Table S3. Variance analysis of regression equation of Box-Behnken design for medium ingredient

Source	Freedom	Sum of squares	Mean square	*F* values	*P* values
X_1	1	0.012325	0.012325	12.98	0.009
X_2	1	0.000741	0.000741	0.78	0.406
X_3	1	0.013861	0.013861	14.59	0.007
X_1X_2	1	0.000576	0.000576	0.61	0.462
X_1X_3	1	0.007056	0.007056	7.43	0.030
X_2X_3	1	0.005700	0.005700	6.00	0.044
X_1^2	1	0.094958	0.094958	99.97	0.000
X_2^2	1	0.023322	0.023322	24.55	0.002
X_3^2	1	0.012018	0.012018	12.65	0.009
Model	9	0.182230	0.020258	21.32	0.000
Pure error	4	0.001467	0.000367		
Lack of Fit	3	0.005182	0.001727	4.71	0.084

Supplementary Table S4. Variance analysis of regression equation of Box-Behnken design for fermentation condition

**Source**	**Freedom**	**Sum of squares**	**Mean square**	***F* values**	***P* values**
X_1	1	0.012325	0.012325	16.07	0.005
X_2	1	0.013861	0.013861	122.88	0.000
X_3	1	0.000741	0.000741	39.76	0.000
X_1X_2	1	0.007056	0.007056	8.09	0.025
X_1X_3	1	0.000576	0.000576	5.29	0.055
X_2X_3	1	0.005700	0.005700	3.45	0.106
X_1^2	1	0.094958	0.094958	21.37	0.002
X_2^2	1	0.012018	0.012018	131.18	0.000
X_3^2	1	0.023322	0.023322	122.33	0.000
Model	9	0.182230	0.020258	55.31	0.000
Pure error	4	0.001467	0.000367		
Lack of Fit	3	0.005182	0.001727	4.26	0.087
